# Microstructure Adjustment of Spherical Micro-samples for High-Throughput Analysis Using a Drop-on-Demand Droplet Generator

**DOI:** 10.3390/ma12223769

**Published:** 2019-11-16

**Authors:** Saeedeh Imani Moqadam, Lutz Mädler, Nils Ellendt

**Affiliations:** 1 Faculty of Production Engineering, University of Bremen, Badgasteiner Straße 1, 28359 Bremen, Germany; s.imani@iwt.uni-bremen.de (S.I.M.); lmaedler@iwt.uni-bremen.de (L.M.); 2 Leibniz Institute for Materials Engineering IWT, Badgasteiner Straße 3, 28359 Bremen, Germany

**Keywords:** cooling rate, droplet cooling, microstructure, drop-on-demand, high-throughput, SDAS, secondary dendrite arm spacing

## Abstract

High-throughput methods for the development of structural materials require samples which are comparable in geometric dimensions and microstructure. Molten metal droplet generators produce thousands of droplets and microspheres from specific alloys with very good reproducibility. In this study, droplet generation experiments were conducted with two alloys and their microstructure was analyzed regarding secondary dendrite arm spacing (SDAS) in order to determine cooling rates during solidification. A droplet cooling model was developed, and predictions showed good agreement with the experimental data. Finally, a sensitivity study was conducted using the validated model to identify critical process parameters which have great impact on the resulting microstructure and need to be well-controlled to achieve the desired reproducibility in microstructure.

## 1. Introduction

High-throughput methods allow evaluating a large number of samples based on their properties such as thermal stability [1], phase composition [2], hardness [3,4], formability [5,6,7,8], machinability [9], or corrosion behavior [10]. The high-throughput methods for structural materials require samples to have a defined microstructural state since it can be as influential as the materials’ composition in determining material properties [11]. Thus, it is vital to generate reproducible samples of the same geometry (diameter and shape) and microstructure. Adjustment of the microstructure is determined by the thermal history of the sample, which depends on solidification and cooling conditions as well as subsequent heat treatment.

The thermal energy of a single metal droplet moving in a cooler fluid is transferred via forced convection and radiation while heat conduction within the droplet can usually be neglected due to low Biot numbers [12]. In order to model the cooling rate and solidification, the coupling of droplet motion and heat transfer should be taken into account.

### 1.1. Droplet Motion

Understanding the movement of a single droplet in a stagnant gas atmosphere is necessary for the examination of the single drop cooling while it solidifies. The single molten metal droplet is geometrically regarded as a sphere due to its very high surface tension. For the free fall of a sphere in a stagnant gas, the following equilibrium of forces can be assumed.

(1) $$F_{i}=F_{g}-F_{b}-F_{D},$$

where: $F_{g}=g.\rho_{p}\frac{\pi }{6}d_{p}^{3}$, $F_{b}=g.\rho_{f}\frac{\pi }{6}d_{p}^{3}$, and $F_{D}=c_{D}.\rho_{f}\frac{\pi }{4}d_{p}^{2}.\frac{v^{2}}{2}$, $F_{i}=\frac{\mathrm{dv}}{\mathrm{dt}}.\rho_{p}\frac{\pi }{6}d_{p}^{3}$. $F_{D}$ is the drag force, $F_{g}$ is the weight force, $F_{b}$ is the buoyancy force and $F_{i}$ is the inertia force. A summary of symbols can be found in the nomenclature section. Equation (1) will result in:

(2) $$\frac{dv}{dt}=g-\frac{3}{4d_{p}}c_{D}.\frac{\rho_{F}}{\rho_{P}}.v^{2}$$

for the evolution of droplet velocity with time. The droplet trajectory can be obtained by integration of Equation (2):

(3) x(t)=∫v(t)dt

There are correlations for different Reynolds numbers to determine the drag coefficient $c_{D}$. For Reynolds numbers between 1 < Re < 800 the correlation according to Schiller–Naumann [13] can be used:

(4) cD=24Re−1(1+0.15Re0.687)

This correlation is commonly applied in spray models [14,15]. Schiller–Naumann’s correlation has been developed for nearly isothermal conditions (droplet surface temperature $T_{s}$, free stream ambient temperature $T_{\infty }$). However, when the surface temperature is much higher than the ambient temperature, the flow field is strongly influenced by the heat transfer between sphere and ambient gas. In the case of non-isothermal conditions, we recently developed a correction coefficient to the Schiller–Naumann’s correlation for non-isothermal conditions [16]:

(5) cD=24Re−1(1+0.15Re0.687)ϕ ; ϕ=0.273(1−0.883Re)(ρ∞ρS−1)+1

The drag coefficient is strongly influenced at high gas velocities due to gas expansion around the droplet. Equation (5) is based on data for 1 < Re < 130 for helium and nitrogen and reaches a coefficient of determination R^2^ equal to that of the original correlation.

### 1.2. Heat Transfer of a Sphere

The heat transfer of a single droplet to the flow is governed by convection and radiation [17]. Convective heat transfer occurs when transport of energy is caused due to movement of a flow. The heat transfer is represented by:

(6) $$d\rho_{p}c_{p}\frac{dT_{p}}{dt}=-6\left[h\left(T_{p}-T_{\infty }\right)+\sigma \epsilon \left(T_{p}^{4}-T_{\infty }^{4}\right)\right]$$

The heat transfer coefficient is determined using the Nusselt equation based on various correlations. For Reynolds numbers smaller than 200, the common correlation of the Ranz–Marshall Model [18] can be used, taking into account that all gas properties are calculated at the film temperature $\left(T_{F}=\frac{T_{S}+T_{\infty }}{2}\right)$:

(7) NuF=2+0.6ReF1/2 PrF1/3

The validity of this correlation for high temperature differences has been recently proved [16].

**Figure 1:**
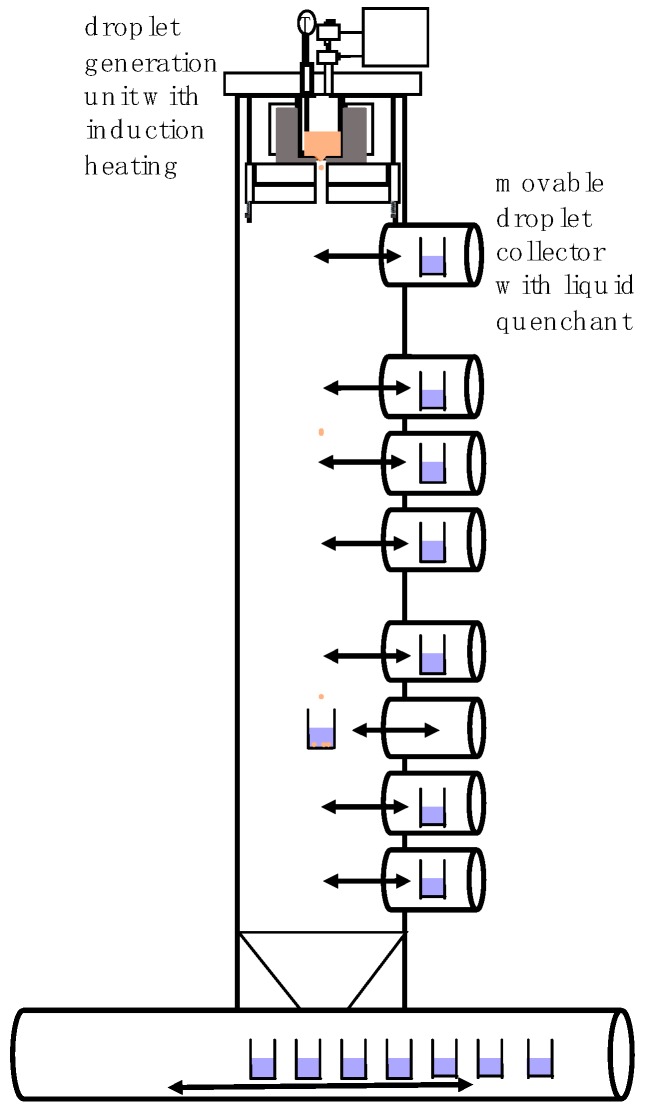
Schematic view of the droplet generator consisting of a droplet generation unit and a tower with movable particle collectors.

In order to model the solidification of a droplet, a simple model corresponding to the definition of cooling rate with its motion has been applied. In the experimental part of the study, the equations correlating the cooling rate and secondary dendrite arm spacing were used. We assume that solid fraction is a linear function between the liquidus and solidus temperatures. Therefore, the release of latent heat *L* can be modeled:

(8) $$c_{p,L\to S}=\frac{L}{T_{L}-T_{S}}$$

for the specific heat for temperatures between solidus and liquidus. From calculated thermal histories, the average cooling rate during solidification is calculated as

(9) $$\dot{T}=-\frac{T_{L}-T_{S}}{t_{L}-t_{S}}$$

where $t_{L}$ and $t_{S}$ are the times when liquidus and solidus are reached, respectively (Figure 1).

### 1.3. Experimental Generation and Analyses of Molten Metal Droplets

The droplet generators are meant to generate droplets of the same size and properties, and have many applications in science and industry such as investigating the droplet behavior, placing solder balls on electronic chips in electronics industries, or droplet-based additive manufacturing techniques [19,20,21]. In the past, we have shown that drop-on-demand droplet generation and solidification of the spheres are possible even for metals with high melting points, such as steels [22]. It has also been demonstrated that correlations for secondary dendrite arm spacing can be used to determine the average cooling rate of molten metal droplets during solidification in sprays [23,24] to obtain a better understanding of process conditions experimentally.

In this study, highly reproducible microsamples of a defined size and microstructure were synthesized using a pneumatic drop-on-demand droplet generator. The samples were generated from two alloys, namely AlCu4.5 and CuSn6. The properties of two alloys are shown in Table A1. Such particles may be used for high-throughput analyses regarding material properties. Compared to other atomization and continuous droplet chain processes, the thermal history of the droplets is not influenced by thermal coupling of neighbored droplets. Secondary dendrite arm spacing (SDAS) analysis of individual particles was performed to obtain their thermal history. Using this method, we generated experimental data of cooling rate for different process conditions which were used to validate a droplet trajectory and cooling model. After validation, we used the model to investigate a sensitivity study of the process parameters in order to evaluate the effect of specific process conditions on the cooling rate and the resulting microstructure. Key parameters can hence be controlled to obtain specific, reproducible microstructures of generated samples for high-throughput methods or other applications.

## 2. Materials and Methods

In the present work, the cooling rate of single metal droplets, generated via the drop-on-demand process, was investigated theoretically and experimentally. For this purpose, experiments were carried out with two different alloys, AlCu4.5 and CuSn6, which have different thermophysical properties. In the experiments, droplets with different diameters were generated and the droplets were further solidified to form spherical particles. In order to model the cooling rate of the particles, their size and velocity were initially taken into account. The Malvern Morphology G3 system (Software Version 8.23, Malvern Panalytical GmbH, 34123 Kassel, Germany) was used to determine the size distribution of the particles and a high-speed camera was applied to determine the initial velocity of the droplet, which was used as the initial boundary condition for the solution of the trajectory in Equation (3).

### 2.1. Experimental Setup

The single droplet generator [22] was mounted in a chamber with a height of 6.5 m and was equipped with different height movable droplet collectors filled with a quenching liquid. It was therefore possible to collect droplets in different states (liquid, semi-solid, or solid). In the upper part of the tower, there was the droplet generation unit in which the feedstock material was molten and droplets were produced. The crucibles were made of ceramic or graphite as required by the melt. During the process the crucible, containing the melt material, was heated up indirectly via an induction furnace. The melt oscillations were avoided by using indirect heating method which resulted in stable droplet generation. The temperature of the melt was controlled using a Type B thermocouple during the whole process and the entire chamber was purged with nitrogen, argon, or helium. A schematic of the droplet generator is shown in Figure 1.

In this study, we used graphite crucibles with a nozzle diameter of 800 µm for AlCu4.5 and 850 µm for CuSn6, and a nitrogen atmosphere. After the melt reached a temperature of about 100 K above its liquidus temperature, the valve connected to a feed pressure was opened for a short time interval (usually 1–5 ms according to melt properties and aimed droplet size) and was switched off afterwards. The procedure was repeated continuously and automatically and introduced a pressure oscillation via the applied pressure pulse. This let the droplet out of the nozzle as the valve opened and detached the droplet as the pressure dropped (valve switched off). Each pressure pulse generated one droplet and when the stable single droplet generation modus was reached, droplets of the same size were released from the nozzle. A high-speed camera was used to record droplet formation, detachment, and initial movement of the generated droplets at a framerate of 2000 fps and a resolution of 39 pixels/mm.

The droplets cooled in the purged atmosphere as they fell along the tower and solidified. After the experiment, the spherical particles were removed from the collectors, cleaned, dried, and prepared for further processing.

### 2.2. High Speed Imaging Analysis

**Figure 2:**
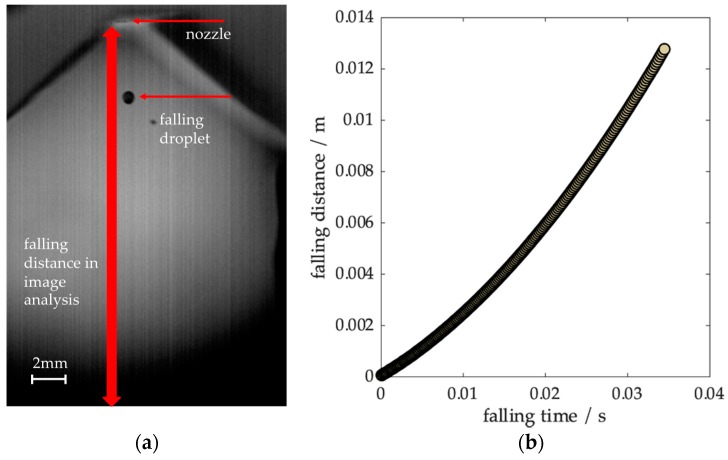
(**a**) Image from a high-speed sequence showing a CuSn6 droplet after detachment. (**b**) The measured droplet trajectory from image analysis.

The image analysis software ImageJ (Version 1.8.0, National Institute of Health, Bethesda, MD 20892, USA) was used to obtain particle positions by image segmentation. An example of an image section and the resulting measured trajectory can be seen in Figure 2. A tangent was calculated at *t* = 0 to determine the initial velocity. For the evaluation, the images of at least four particles per process parameter were evaluated.

### 2.3. Microstructural Parameters

The solidification microstructures of a pure metal or an alloy fall mainly into two groups: Single-phase primary crystals and polyphase structures [25]. The most important growth form is the dendritic growth which is formed as a tree-like primary crystal. The dendrites’ size and shape could be used to characterize the solidification process. When a dendritic structure forms, the dendrite arms grow parallel to the favorable growth directions controlled by a diffusion-limited process [26]. During growth, the secondary dendrite arm spacing decreases with increasing cooling rate.

In order to estimate the average cooling rate for a given microstructure, initially the total length *d* [µm] from the first to the last secondary arm was measured and the number of secondary arms *n* was determined. The SDAS is the distance between the first adjacent arm to the last, divided by the number of arms minus one, as follows:

(10) $$SDAS=\frac{d}{n_{arms}-1}.$$

The cooling rate can be calculated via the following empirical equation:

(11) $$\lambda =\lambda_{0}CR^{-n},$$

where CR is the cooling rate, $\lambda_{0}$ is constant, and the exponent n ranges from 0.2 to 0.4 [27,28]. Equation (11) was used to determine the cooling rate of the alloys AlCu4.5 and CuSn6. As $\lambda_{0}$ and n are material-dependent constants, it applies to AlCu4.5 according to Kirkwood et al. $\lambda_{0}$ = 50 and n = 1/3 [29]. For CuSn6, according to Choi et al., $\lambda_{0}$ = 34 and n = 0.323 [27].

To prepare the particles for the experimental cooling rate analysis, they were embedded in an epoxy resin for each set of process parameters, grinded, and polished. The particles were etched with the parameters described in Table 1.

**Figure 3:**
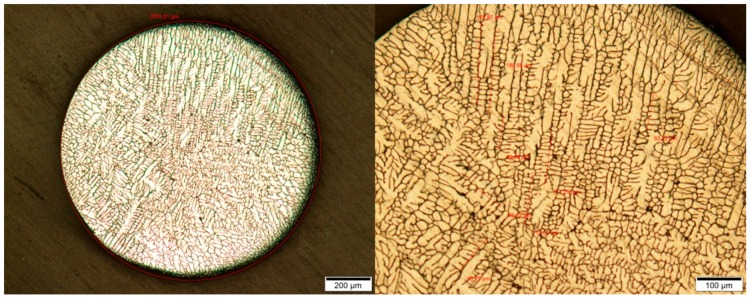
Polished and etched particle of AlCu4.5 at two different magnifications of 5× (left) and 10× (right). Particle diameter: 978 µm.

From each parameter, 10 droplets were analyzed by measuring at least 10 dendrites using the light microscope Olympus BX51. Figure 3 shows an exemplary etched particle of AlCu4.5 at two magnifications. The dendritic structure with dendrites growing in all directions—as expected in the cooling processes without containers—is clearly visible.

### 2.4. Droplet Cooling Model

Equations (2) and (6) were solved in Matlab simultaneously to obtain the droplet trajectory and thermal history. The average cooling rate was then determined using Equation (9). Finally, the SDAS correlation presented before was used to predict secondary dendrite arm spacing.

## 3. Results

### 3.1. Initial Velocity

**Figure 4:**
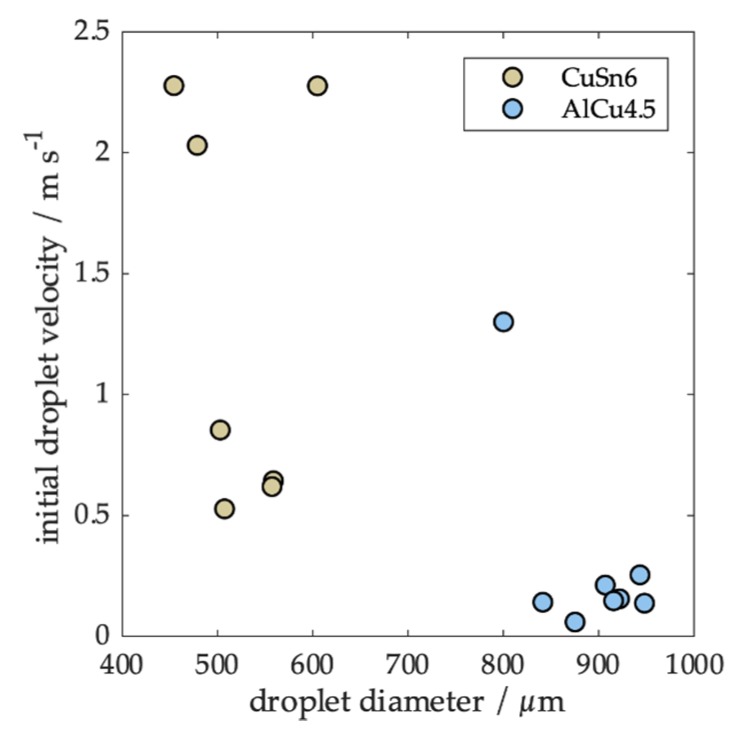
Measured initial velocities for both alloys. A minimum velocity is achieved at the change from ejection to jetting droplet formation mode.

Droplets were produced at different droplet generation conditions via the drop-on-demand process (a detailed list of process conditions can be found in Table A2 and Table A3). Small droplets were generated in an ejection mode as they were formed inside the nozzle. The droplet formation mode changed to the jetting mode when the formation time was increased. In the jetting mode, a droplet was separated from a ligament outside the nozzle via inertia forces [22]. As a result, bigger droplets were formed. Since more energy was needed to create the surface energy of the ligament, less energy was available for the initial kinetic energy of the droplet. This effect is clearly visible in Figure 4 for the CuSn6 droplets, while most of the aluminum droplets are close to the minimum. As a result, we were able to analyze droplets of similar sizes, but with a very different initial velocity to study its effect.

### 3.2. Secondary Dendrite Arm Spacing and Experimentally Determined Cooling Rate

**Figure 5:**
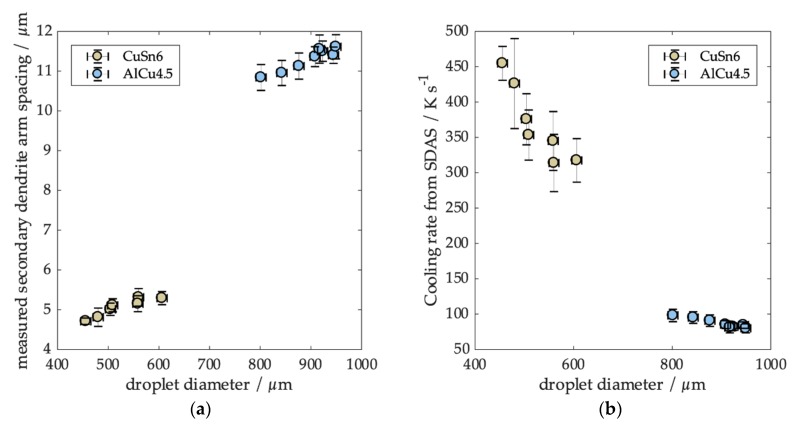
(**a**) Measured secondary dendrite arm spacing (SDAS) and (**b**) the resulting cooling rate as a function of droplet diameter.

The results from SDAS analysis based on particle diameter are illustrated in Figure 5a. Each point represents the mean SDAS for 10 particles with the same diameter. For the AlCu4.5 particles, the SDAS ranges between 11.02 and 11.86 µm for particle diameters between 802 and 949 µm, respectively (AlCu4.5). For CuSn6, SDAS increases from 4.62 µm to 5.27 µm for particle diameters from 456 to 606 µm. For both alloys, we obtained a clear trend. Cooling rates obtained from SDAS are shown in Figure 5b. We obtained a cooling rate range between 300 and 450 Ks^−1^ for CuSn6 and from 80 to 100 Ks^−1^ for AlCu4.5. This allowed us to compare data in a wide range of cooling rates at different temperature levels with the predictions from the cooling rate model.

### 3.3. Modeling of Evolution of Particle Temperature and Velocity over Falling Distance/Time

**Figure 6:**
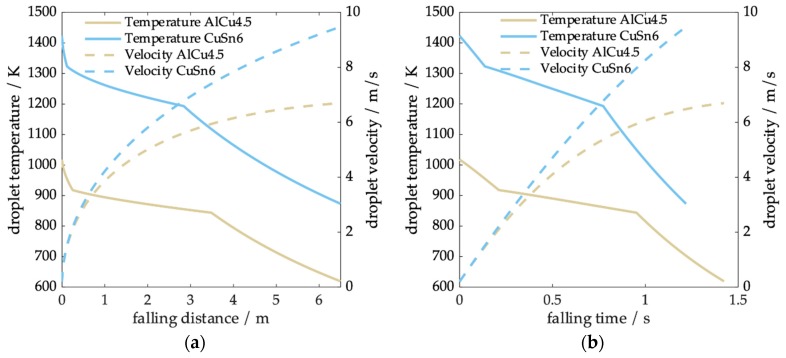
Evolution of particle temperature and velocity as a function of (**a**) falling time and (**b**) falling distance for a droplet diameter of 800 µm.

In Figure 6, the temperature and velocity are plotted as a function of falling distance (Figure 6a) and falling time (Figure 6b) for an AlCu4.5 and a CuSn6 particle (d_p_ = 800 µm). The cooling rate was calculated with an initial velocity of 0.2 ms^−1^ and a superheat temperature of 100 K. The particles fell all along the tower and therefore had a maximum falling distance of 6.5 m. Inspection of the figure indicates that the CuSn6 droplets accelerate and fall faster due to the higher density and achieve a higher terminal velocity. Consequently, the velocity of the CuSn6 droplet still rises strongly, while the AlCu4.5 particle velocity slowly merges into the plateau. Accordingly, the fall time with CuSn6 was approximately 0.2 s shorter. The thermal history clearly showed the solidification between liquidus and solidus temperature. For both alloys, the solidification time was similar. While latent heat of AlCu4.5 was about double that of CuSn6 and its specific heat was almost three times higher than that of CuSn6, its density was about 3.5 times lower and the temperature level during solidification was much lower. This leads to a decreased driving temperature difference for both convection and radiation and a lower convective heat transfer coefficient due to changes in gas properties with temperature. Since droplet velocities were higher for CuSn6, their solidification distance was slightly lower.

### 3.4. Comparison of Experimental and Modeling Data

**Figure 7:**
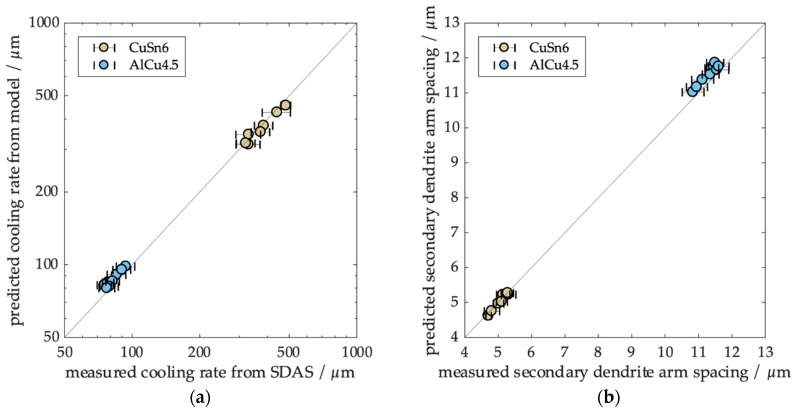
Comparison of the theoretical and experimental determination of (**a**) cooling rate and (**b**) secondary dendrite arm spacing.

In order to obtain a holistic picture of the solidification of the droplets, thermal histories were calculated for each experimental condition based on the determined droplet diameter, melt temperature, and initial velocity. From the thermal histories, average cooling rates during solidification and secondary dendrite arm spacing prediction were determined. Figure 7 summarizes the results of the comparisons. A good agreement between experimental and theoretical results was achieved for both cooling rates and secondary dendrite arm spacing. We conclude that the model gives valid results in this range of process conditions.

## 4. Discussion

In this work, we have conducted experiments on the generation and solidification of single droplets. We experimentally determined the cooling rates and compared them with the prediction from a single droplet cooling model. To summarize the influence of specific process parameters and the influence of possible variations on the reproducibility, we conducted a computational sensitivity study. We assumed a standard set of parameters consisting of a droplet diameter of 800 µm, an initial velocity of 0.8 ms^−1^ for AlCu4.5 and 1.5 ms^−1^ for CuSn6 (according to average velocities from Figure 4), and a melt superheat of 100 K. Based on these parameters, we determined the percental change in the cooling rate for AlCu4.5 and CuSn6 for individually varied parameters.

**Figure 8:**
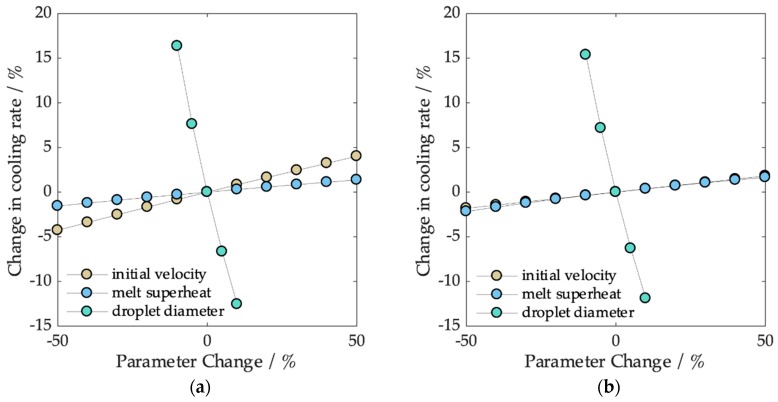
Sensitivity of the cooling rate based on process parameters (initial velocity, melt superheat, and droplet diameter) for (**a**) CuSn6 and (**b**) AlCu4.5.

The sensitivity of the cooling rate is demonstrated in Figure 8. As can be seen, changes of up to +/− 50% in the initial velocity (v_0_) affected the cooling rate by 2% for AlCu4.5 and 5% for CuSn6. This is because CuSn6 droplets have a much higher terminal velocity due to their higher density, while relative changes have less effect for AlCu4.5 particles which are slower. The convective heat transfer coefficient was not strongly affected by such small velocity changes. The same percental changes in melt superheat led to a change in the cooling rate of 2% for both alloys. Here, the effect is that for an increased melt superheat, solidification will start after the droplets have accelerated to a higher velocity, which again slightly increases the convective heat transfer coefficient.

The droplet diameter on the other hand, has a major influence on the cooling rate, a result which is also physically expected as it is directly related to the surface-to-volume ratio. A small change in diameter may result in a 15 percent or more change in the cooling rate. A change of droplet diameter by 5% (equal to +/− 20 µm for a diameter of 800 µm) leads to a change of approximately 7% in the cooling rate for both alloys.

Droplet diameter and initial velocity are coupled by the droplet formation mode, meaning that a high reproducibility in the droplet diameter will also lead to a high reproducibility in initial droplet velocity (see Figure 4, CuSn6). During the experiments, the temperature control of the melt was to achieve within +/− 10 K, resulting in a parameter change of 10% with almost no effect on the cooling rate.

## 5. Conclusions

The cooling rate of spherical particles made out of two different alloys AlCu4.5 and CuSn6 was analyzed and calculated both experimentally and theoretically. The particles from the above materials with melting temperatures of various ranges were generated using the pneumatic drop-on-demand droplet generator. The experimental cooling rate was determined via analyzing the microstructure and measuring the secondary dendrite arm spacing. Using the empirical equations based on the microstructure, the cooling rate was determined experimentally and compared to those obtained from a model that we developed for single droplet cooling. We used corrected Schiller–Naumann correlations for high temperature differences to determine the drag coefficient and the Ranz–Marshall correlation to calculate the Nusselt number. We obtained a validation of our model for cooling rates between 80 and 450 Ks^−1^ for AlCu4.5 and CuSn6. The validated model can be used for calculating the cooling rate of many other alloys in the melting ranges up to 1600 °C. Together with high reproducible droplet generation and individual droplet cooling, we developed a method to obtain fundamental data regarding the dependency of microstructure on the cooling rate of novel materials. Regarding the application of drop-on-demand droplet generation and solidification as a synthesis process for high-throughput methods, we showed that droplet diameter coupled with its initial velocity were the most influential parameters on the resulting microstructure. High reproducibility in droplet formation is the strength of droplet generators. We therefore conclude that this process is a versatile tool to produce thousands of samples in a short time from various alloys for analyses in high-throughput methods.

## Figures and Tables

**Table 1:** Etchant recipes and etching time.

Alloy	Etchant	Etching Time (s)
AlCu4.5	10 g NaOH + 90 g H_2_O	40
CuSn6	10 g (NH_4_)_2_S_2_O_8_ + 100 g H_2_O	90

## Nomenclature

**Greek Symbols**	
*λ*	Thermal conductivity of the fluid, W m^−1^ K^−1^
*λ*_0_	Constant for determining the cooling rate using SDAS
*ρ_F_*	Density of the fluid, kg/m^3^
*ρ_P_*	Density of the particle, kg/m^3^
**Roman Symbols**	
*A_P_*	Particle surface, m^2^
*c_P_*	Heat capacity of the particle, J kg^−1^ K^−1^
*d_P_*	Particle diameter, m
*F_b_*	Buoyancy force, N
*F_g_*	Weight force, N
*F_i_*	Inertia force, N
*F_D_*	Drag force, N
*g*	Gravitational acceleration, m s^−2^
*m_P_*	Mass of the particle, kg
t	Time, s
T_F_	Fluid temperature, K
T_L_	Liquidus temperature, K
T_M_	Melt temperature, K
T_s_	Surface temperature, K
T_film_	Film temperature, K
v	Velocity, m s^−1^
**Non-dimensional numbers**	
$Nu=\frac{hd}{k}$	Nusselt number
*Nu_F_*	Nusselt number at film temperature
$Pr=\frac{\eta c_{p}}{\lambda }$	Prandtl number
*Pr_F_*	Prandtl number at film temperature
*Pr*_∞_	Prandtl number at ambient temperature
$Re=\frac{ud\rho }{\eta }$	Reynolds number
*Re_F_*	Reynolds number at film temperature
*c_D_*	Drag coefficient
*n*	Constant for determining the cooling rate using SDAS
*n_arms_*	Number of secondary dendrite arms
**Abbreviations:**	
fps	Frames per second
SDAS	Average secondary dendrite arm spacing, µm
CR	Cooling rate

## Appendix A. Overview of Material Properties and Experimental Parameters During the Droplet Generation

**Table A1:** Properties of AlCu4.5 and CuSn6 [30,31].

Alloy	Specific Heat Capacity (J/gK)	Latent Heat (J/kg)	Density (g/cm^3^)	Liquidus Temperature (°C)	Solidus Temperature (°C)
**AlCu4.5**	1.148	381.774	2350	644.8	571
**CuSn6**	0.394	187.890	7948	1050	900

**Table A2:** Solidification conditions and obtained values for SDAS and cooling rate for CuSn6.

Droplet Diameter µm	Initial Velocity m s^−1^	Measured SDAS µm	Standard Deviation of SDAS µm	Cooling Rate from SDAS K s^−1^	Cooling Rate Prediction from Model K/s	SDAS Prediction from Model µm
560	0.638	5.31	0.44	313.7	330.6	5.22
559	0.614	5.15	0.4	344.9	331.3	5.22
504	0.848	5.01	0.31	375.6	386.4	4.96
456	2.272	4.71	0.16	454.7	484.2	4.62
606	2.271	5.29	0.33	317.4	321.6	5.27
509	0.522	5.11	0.33	353.3	374.2	5.02
480	2.025	4.81	0.46	426.1	442.8	4.75

**Table A3:** Solidification conditions and obtained values for SDAS and cooling rate for AlCu4.5.

Droplet Diameter µm	Initial Velocity m s^−1^	Measured SDAS µm	Standard Deviation of SDAS µm	Cooling Rate from SDAS K s^−1^	Cooling Rate Prediction from Model K/s	SDAS Prediction from Model µm
802	1.295	10.84	0.65	98.1	93.9	11.02
945	0.249	11.40	0.41	84.4	78.3	11.71
923	0.15	11.5	0.51	82.2	75.3	11.86
877	0.054	11.13	0.66	90.8	85.5	11.37
843	0.136	10.95	0.63	95.1	90.3	11.16
908	0.207	11.36	0.5	85.2	82.0	11.52
917	0.142	11.55	0.71	81.0	79.2	11.66
949	0.132	11.61	0.61	79.9	77.3	11.75
